# Pubertal Timing and Health-Related Quality of Life—A Cross-Sectional Study of Polish Adolescents

**DOI:** 10.3390/pediatric17030069

**Published:** 2025-06-18

**Authors:** Zbigniew Izdebski, Alicja Kozakiewicz, Katarzyna Porwit, Michalina Aleksandra Gryglewska, Joanna Mazur

**Affiliations:** 1 Department of Biomedical Aspects of Development and Sexology, Faculty of Education, University of Warsaw, 00-561 Warsaw, Poland; zbigniew.izdebski@uw.edu.pl; 2 Department of Humanization of Medicine and Sexology, Collegium Medicum, University of Zielona Gora, 65-046 Zielona Gora, Poland; a.kozakiewicz@inz.uz.zgora.pl; 3 Centre of Migration Research, University of Warsaw, ul. Pasteura 7, 02-093 Warsaw, Poland; katarzyna.porwit@uw.edu.pl; 4 Collegium Medicum, University of Zielona Gora, 65-046 Zielona Gora, Poland; michalinagryglewska@gmail.com

**Keywords:** pubertal timing, health-related quality of life (HRQL), adolescents, psychological well-being, KIDSCREEN-27, cross-sectional study, Poland

## Abstract

**Background/Objectives:** In research on the relationship between pubertal timing and adolescent health, more attention is typically given to early rather than late maturation, as well as the associated risk of engaging in health-compromising behaviors. The aim of this study was to assess changes in HRQL (health-related quality of life) depending on subjectively perceived pubertal timing, measured in five categories. **Methods:** A cross-sectional online survey was conducted in spring 2024 in a western region of Poland (*N* = 9411; mean age 15.15 ± 1.56 years). Mean KIDSCREEN-27 index scores were compared according to self-reported pubertal timing, and five relevant general linear models were estimated, adjusting analyses for respondents’ age, sex, and the remaining four HRQL scores. **Results:** In the study group, 49.0% of students assessed their pubertal timing as typical, 28.5% as earlier, and 22.5% as later compared to peers of the same sex. For all five KIDSCREEN-27 dimensions, adolescents who matured at a pace perceived as typical achieved the highest quality-of-life index scores. Significantly earlier or significantly later pubertal timing was associated with a notable decrease in these indices. Some significant interactions were identified between sex or age and pubertal timing as predictors of HRQL. The strongest association with pubertal timing was observed for the Psychological Well-being dimension, where differences unfavorable to older age groups were additionally linked to delayed pubertal timing. **Conclusions:** Greater awareness of the relationship between perceived pubertal timing and adolescents’ well-being is warranted among preventive care physicians, parents, and school psychologists and educators.

## 1. Introduction

Adolescence is a pivotal transitional stage between childhood and adulthood, marked by profound biological, psychological, and social changes. Human maturation is shaped by both biological and social influences, including the family and peer environments, as well as hormonal, anatomical, and physiological changes [1]. Differences in behaviors and reactions linked to pubertal timing may present challenges not only for parents and teachers but also, above all, for adolescents themselves [2]. Drawing on the theory of maturational asynchrony proposed by the eminent Polish sexologist Andrzej Jaczewski, we recognize that biological development does not necessarily occur at the same pace as social or psychological development [3]. In light of this, a key question is how these factors—and the mismatch in the timing of different aspects of development—contribute to the psychological changes experienced by adolescents and shape their overall quality of life.

From the perspective of biological development, puberty marks the attainment of biological maturity, defined as the body’s physical ability to reproduce. Pubertal development is commonly assessed using the five-stage Tanner scale, which evaluates breast development in girls, and the development of the testes, penis, and scrotum in boys, as well as pubic hair growth in both sexes [4]. Precocious puberty refers to the appearance of secondary and tertiary sexual characteristics before the age of 8 in girls and before the age of 9 in boys. Typical indicators of normally progressing puberty include the onset of menarche in girls and the first nocturnal emission in boys. A study involving 71,341 women found that the average age of menarche was 12.5 years among those born between 1950 and 1969, and 11.9 years among those born between 2000 and 2005. These findings suggest that over the span of 55 years, the average age of menarche has declined. Furthermore, the percentage of women who experienced early menarche (before age 11) rose from 8.6% to 15.5%, while the share experiencing very early menarche (before age 9) increased from 0.6% to 1.4% [5]. Similar trends have been reported in Poland, where researchers have observed a gradual decline in the age at first menstruation among girls over time [6]. This phenomenon is particularly relevant in the context of the aforementioned theory of maturational asynchrony. However, unlike in the case of girls, there is a notable lack of equally detailed research tracking changes over time in the age of first nocturnal emission in boys. There are also some studies suggesting that the COVID-19 pandemic period may have been conducive to precocious puberty. These were mostly based on data from Asian countries or Italy and mainly concerned girls [7,8].

Delayed puberty, on the other hand, is defined as the absence of signs at an age at least two standard deviations above the average age for the onset of puberty in a given population. In Poland, delayed puberty is diagnosed in girls if breast development has not begun by age 13 and in boys if the testes have not enlarged beyond 4 mL by age 14, or if full sexual maturity is not reached within 4–4.5 years from the onset of puberty (marked by the occurrence of menarche in girls and nocturnal emissions in boys) [9].

The duration of puberty therefore varies between individuals; however, it is important to consider not only its objective indicators but also how it is perceived by the individual. Pubertal timing refers to the stage at which an individual progresses through the various phases of development compared to their peers [10,11]. Peer groups can play a vital role in how adolescents adapt to the changes brought on by puberty and serve as an important context in which attitudes toward the body are formed. This is especially important because adolescents tend to develop their beliefs and habits through daily interactions with their immediate surroundings, including family, school, peers, and other social groups (e.g., hobby-related communities) [12].

Studies have found early puberty to be linked to more frequent use of psychoactive substances such as alcohol, nicotine, and cannabis, as well as to depressive disorders, antisocial behavior, and risky sexual behavior [13]. A 2020 review found that early pubertal timing in girls was associated with poorer relationships with family and peers, lower mood, and depression. Among boys, early pubertal timing was related to increased depressive symptoms, externalizing behaviors, and deteriorated social relationships [14], as confirmed by a meta-analysis by Dimler and Natsuaki [15], which found a small but significant effect of early maturation on externalizing behaviors.

Girls who matured at an average or later age than their peers reported better overall health and were less likely to report feeling unwell compared to girls who matured early [16]. Later puberty in girls was also associated with a shorter duration of pubertal development [17]. A 2020 study suggests that among 16-year-old boys, delayed puberty is linked to symptoms of depression [18]. However, there is a limited body of research on delayed puberty, particularly among boys, highlighting the need for further studies in this area.

In research on health-related quality of life (HRQL), a central issue is defining the concept clearly and choosing an appropriate method of measurement. According to the World Health Organization, quality of life refers to an individual’s subjective assessment of their position in life, viewed in the context of cultural norms, value systems, social relationships, and their personal goals, expectations, standards, and interests [19]. A variety of generic instruments exist for measuring HRQL in children and adolescents. These include the KIDSCREEN group of tools, developed by an international team of researchers from 13 countries in the early 2000s [20]. These questionnaires have since been translated into more than 40 languages (www.kidscreen.org; accessed on 9 April 2025). The KIDSCREEN-27 instrument provides a way to explore selected factors affecting the HRQL of today’s youth in a Central European context [21].

Overall, the current literature reveals a gap in our understanding of the relationship between pubertal timing and quality of life among children and adolescents. It is therefore especially important to investigate whether very early or very late puberty—depending on the respondent’s sex and age—can influence psychosocial functioning. As such, the aim of this study was to assess the extent to which subjectively perceived pubertal timing affected variation in quality-of-life indices among Polish children and adolescents from schools in a single Polish region (the Lubuskie Voivodship), as measured by the KIDSCREEN-27 questionnaire. It was hypothesized that boys and girls who assessed their puberty timing as deviating from the norm for same-sex peers would rate their overall well-being worse as measured by various dimensions of HRQL. The following research questions were formulated:

(1) Does the worsened assessment of quality of life occur for both puberty timing perceived as earlier and later as compared to peers?
(2) For which dimensions of HRQL are the differences between typical and atypical subjective puberty timing the greatest?
(3) Is the association between perceived puberty timing and HRQL subindices observed in both sexes?
(4) Does the significance of the relationship between HRQL and perceived puberty timing and the strength of this relationship change with age?

## 2. Materials and Methods

### 2.1. Participants and Procedures

An anonymous online survey was conducted between March and May 2024 in the Lubuskie Voivodeship, a region of western Poland situated near the border with Germany. Invitations were sent out to all primary and secondary schools in the voivodeship, with a recommendation to survey at least one class from each of three targeted grade levels (Grade 7—approximately age 13; Grade 9—approximately age 15; Grade 11—approximately age 17). The selected age groups were in line with the protocol of the Polish HBSC 2022 study in view of the other planned comparative analyses [22]. The questionnaires were completed during regular class hours. School management and staff were provided with a set of documents to support the study, including written instruction, and ongoing consultation with the research team was provided.

There were 11,903 entries into the web data collection system, of which 1583 were filled outside school hours, completely blank, or incomplete. Finally, data obtained from 9411 students were eligible for analysis. The primary criteria for inclusion were presence at school on the day of the survey of the class in which the student was enrolled and his or her consent to participate in the survey. Records were excluded from the final database if no class level or year of birth was given, if the student’s age differed from that typical for the grade, if the location of the school was given as outside the Lubuskie region, if the questionnaire was completed for less than 10 or more than 60 min, and if there was more than 20% missing data in 110 key variables. Specific school addresses or the type of secondary school (general vs. vocational) were not recorded; only general locations were available. The latter confirmed that this study covered all 14 counties (powiats) in the region. The number of effective cases analyzed varied slightly depending on the type of analysis due to missing data regarding pubertal timing (82 cases—0.9%) and regarding the level of the KIDSCREEN-27 indices (89 to 264 missing cases depending on the sub-index, i.e., a maximum of 2.8% for the psychological well-being dimension).

It is estimated that the 9411 respondents represented around 30% of all school students from the selected age groups in the Lubuskie Voivodeship. The sample of students surveyed can therefore be considered representative of the region. Detailed information on the study design is available in the report by Mazur & Izdebski [22]. The average age of respondents was 15.15 years (*SD* = 1.56), with ages ranging from 12.25 to 19.25 years. Among the participants, 2029 (21.6%) lived in the region’s two largest cities, 4003 (42.5%) in smaller towns, and 3379 (35.9%) in rural areas. Students from the oldest grade were less represented (Grade 7—41.1%; Grade 9—39.0%; Grade 11—21.8%). Boys made up a smaller proportion of the sample than girls (boys—45.5%; girls—54.5%), and the proportion of girls increased across the three grades (51.3%, 56.4%, and 57.2%; *p* < 0.001). The average ages for each grade level were 13.79 years (SD = 0.39), 15.20 years (*SD* = 0.57), and 17.67 years (*SD* = 0.53), indicating relatively small differences in age within each group. In subsequent analyses, the school grade was used as an age category, as it allowed for meaningful comparisons among peers within the same educational level.

### 2.2. Tools

The question on subjectively perceived pubertal timing had been tested previously, as an optional item in the 2005/06 Health Behaviour in School-aged Children (HBSC) study, and it was selected for inclusion in the Polish questionnaire used in the current regional study. Students were asked “Do you think your physical development is any earlier or later than most other boys/girls your age?” with the following response options: “Much later,” “A bit later,” “About the same,” “A bit earlier,” and “Much earlier.” Longitudinal studies have shown that this type of subjective assessment correlates well with objective measures of the pubertal growth spurt (Peak Height Velocity). However, it has also been noted that the question is less reflective of current developmental status, particularly among older adolescents (“measures of perceived pubertal timing should not be interpreted as actual timing, especially if obtained during the early adolescence years when adolescents are in the midst of their pubertal development” [23]). A total of 9329 students responded to the question on pubertal timing. Sex was recorded via self-report, with response options corresponding to biological sex (male/female).

The KIDSCREEN-27 questionnaire is a widely used tool for measuring health-related quality of life, serving as a shortened version of the full KIDSCREEN-52 [24,25]. The KIDSCREEN instruments were developed over 20 years ago, simultaneously in 13 countries and in 10 languages, including Polish. This guarantees good psychometric properties avoiding the pitfalls of linguistic and cultural adaptation.

These instruments were designed for the use of children and adolescents aged 8–18, who can either complete the questionnaire themselves or have it completed by a parent on their behalf. Participants are instructed to respond with reference to the previous week. Most items are rated by the frequency of various experiences (1 = “Never,” 2 = “Seldom,” 3 = “Quite often,” 4 = “Very often,” 5 = “Always”), and less commonly by their intensity (1 = “Not at all,” 2 = “Slightly,” 3 = “Moderately,” 4 = “Very,” 5 = “Extremely”). Only one item—self-rated health—uses a different response scale (1 = “Excellent,” 2 = “Very good,” 3 = “Good,” 4 = “Fair,” 5 = “Poor”). Five questions require reverse scoring. KIDSCREEN-27 results are indexed across five dimensions: Physical Well-Being (5 items), Psychological Well-Being (7 items), Autonomy and Parent Relations (7 items), Peers and Social Support (4 items), and School Environment (4 items). For the analyses, scores were standardized to a 0–100 scale, with higher scores indicating more favorable outcomes. For each of the KIDSCREEN-27 dimensions, in turn, more favorable outcomes entail that the adolescent can be described as follows [26]:

- Physical Well-Being: healthy, physically active, fit, and full of energy;
- Psychological Well-Being: satisfied with life, emotionally balanced, and with a positive attitude toward themselves and the world;
- Autonomy and Parent Relations: maintaining good relationships with parents, feeling supported and valued, having age-appropriate autonomy, and living in a financially stable home environment;
- Peers and Social Support: accepted and supported by peers, with meaningful and reliable friendships;
- School Environment: positive experiences at school, good academic performance, and a sense of belonging in the school environment.

In the collected dataset, the KIDSCREEN-27 indices demonstrated good reliability, with Cronbach’s alpha coefficients of 0.809, 0.860, 0.855, 0.868, and 0.766, respectively. The unidimensionality of the three sub-scales was confirmed, with the exception of the Psychological Well-Being and Autonomy and Parent Relations scales. The percentage of variance explained by the first principal component ranged from 53.3% to 71.9% for the individual KIDSCREEN-27 sub-scales, and the eigenvalue of the second component in the two non-homogenic scales mentioned above was small (1.15 and 1.14, respectively). A more detailed validation of this tool, also taking into account convergent and discriminant validity, was previously conducted during the development phase, also using data from Poland [24,25].

### 2.3. Statistical Analysis

Preliminary analyses checked whether the KIDSCREEN-27 subscales maintained good psychometric properties in the collected sample. Cronbach’s reliability coefficient and principal component analysis (PCA) was applied. Categorical data were presented as counts and percentages. The normality of the distribution of the KIDSCREEN-27 indices was assessed using the Kolmogorov–Smirnov (KS) test, measures of skewness, and kurtosis, as well as graphically using Q-Q plots. For the KIDSCREEN-27 indices, mean values and standard deviations were calculated using the 0–100-point standardized scale. The distribution of responses to the pubertal timing question was compared across groups defined by sex and age using the chi-square test. When interpreting differences, adjusted standardized residuals were considered, with an absolute value greater than 2 indicating a statistically significant difference. Mean KIDSCREEN-27 subindices were also compared according to sex, grade, and pubertal timing categories using the Mann–Whitney or Kruskal–Wallis (KW) test for independent samples. All pairs of groups differing in pubertal timing were compared using a post hoc test. In the final stage of the analysis, five general linear models (GLMs) were estimated to assess the significance of pubertal timing in explaining the variability of the five KIDSCREEN-27 indices, with analyses adjusted for the respondents’ age and sex, as well as other four HRQL indices as covariates. Pairwise comparison was repeated on the basis of the GLM multivariable model. The F-statistic was calculated to compare the variance explained by the contrasts (defined by linear combinations of the regression coefficients) to the unexplained variance (error). A low *p*-value suggested that, at least in one case, students classified into distinct pubertal timing categories differ significantly in terms of their HRQL scores.

Attention was given to the significance of the main effect, as well as to two-way interactions between pubertal timing and the sex/age/remaining four KIDSCREEN-27 scores. Selected significant interactions were graphically presented as marginal means derived from the above GLMs. The goodness-of-fit of the GLMs was analyzed on the basis of the R-square coefficient.

## 3. Results

### 3.1. Distribution of KIDSCREEN-27 Indices in Polish Adolescents

The adolescents surveyed rated their quality of life highest in the dimension of Autonomy and Parent Relations and lowest in the School Environment dimension.

Inferring the normality of the distributions of the individual indices depends on the adapted criteria. The K-S test indicates the non-normality of all distributions with a significance level of *p* < 0.001. The skewness coefficients are close to zero for the Physical Well-Being and School-Environment dimensions and reach negative values from −0.319 to −0.194 for the other three dimensions. The kurtosis coefficients take values from −0.524 (Physical Well-Being) to −0.233 (Autonomy and Parent Relations). On the contrary, according to the graphical method supporting the inference of a normal distribution (Q-Q plot), the points fell along a straight line, with only slight deviations, more pronounced for low values of all indices.

Table 1 displays the mean KIDSCREEN-27 indices by gender and grade. No gender-related differences emerged for the Peers and Social Support dimension. For the other HRQL dimensions, boys achieved significantly higher mean index values than girls. The differences were greatest for the Physical Well-Being dimension and relatively smaller (but already significant) for the School Environment dimension. Age-related differences were confirmed for all dimensions of the KIDSCREEN-27 with *p* < 0.001. For the first three dimensions (Table 1), this meant a systematic deterioration of quality of life with age, while no linear changes were confirmed for the subsequent two dimensions. Regarding the last two dimensions, HRQL scores improved in the middle group against the youngest group. Students of the third year of secondary school had the least favorable results for all five KIDSCREEN-27 dimensions.

A significant correlation was found between the indices of the individual KIDSCREEN-27 quality of life dimensions. The corresponding Pearson coefficients ranged from 0.299 (Peers and Social Support and School Environment) to 0.536 (Physical Well-Being and Psychological Well-Being), indicating moderate strength of the association. A detailed summary of correlation coefficients can be found in the electronic Supplementary Materials (Table S1).

### 3.2. Perceived Pubertal Timing in Polish Adolescents

Table 2 presents data on self-perceived pubertal timing by sex and age of the surveyed students.

Nearly half of all respondents (49.0%) rated their pubertal timing as similar to that of their same-sex peers. According to the adjusted standardized residuals, this percentage was significantly higher among girls than boys. Conversely, boys reported more frequently than girls that they had matured, or were maturing, later than their peers (either “Much later” or “A bit later”). A similar percentage of boys and girls rated their pubertal timing as “A bit earlier,” whereas girls more frequently chose the “Much earlier” response than boys did.

The age groups were also significantly differentiated in subjective assessments of pubertal timing. The 13-year-old group stood out most clearly, being much less likely to rate their pubertal timing as similar to that of their same-sex peers. On the other hand, they were more likely to perceive themselves as maturing either much later or much earlier. No significant differences were observed between the 15- and 17-year-old groups in the distribution of responses to the pubertal timing question (*p* = 0.782).

Perceptions of the pubertal timing for boys and girls were not confirmed to have a statistically significant relationship with the place of residence, so the exact figures are not presented (boys: chi-sq.= 6.39; *p* = 0.604 and girls: chi-sq. = 10.12; *p* = 0.257, respectively).

### 3.3. KIDSCREEN-27 Indices According to Pubertal Timing in the Univariate Analysis

Table 3 shows the mean KIDSCREEN-27 indices in the groups distinguished by perceived pubertal timing. Across all five KIDSCREEN-27 dimensions, adolescents who perceived their pubertal timing as similar to their peers reported the highest HRQL scores. The lowest scores were observed in groups with pronounced deviations in perceived pubertal timing—both much earlier and much later than their peers (Table 2). The Kruskal–Wallis H statistic reached its highest value for the Psychological Well-Being dimension and the lowest for Physical Well-Being, indicating the strongest and weakest effects of pubertal timing, respectively.

The parametric ANOVA test for the comparison of the five groups distinguished by pubertal timing perception confirms the differences between groups shown by the non-parametric method. For the subsequent KIDSCREEN-27 dimensions, the eta-squared coefficients are 0.005; 0.024; 0.019; 0.008; and 0.015, respectively.

Post hoc KW test analysis showed that the KIDSCREEN-27 subscale scores for adolescents who reported typical pubertal timing differed significantly from the scores of all four groups who reported either earlier or later maturation (Table S2). However, the final conclusion on the difference between the groups distinguished by their perception of puberty needs to be corrected for other factors, especially sex and biological age.

### 3.4. KIDSCREEN-27 Indices According to Pubertal Timing in the Multivariate Analysis

In three general linear models, a statistically significant association (*p* < 0.001) was confirmed between the KIDSCREEN-27 indices and subjectively perceived pubertal timing, after adjusting for respondents’ sex and age, as well as for other HRQL indices as covariates.

The association of pubertal timing with variation in the Physical Well-Being and Peers and Social Support indices was not confirmed by multivariate analysis. Furthermore, for the School Environment index, slightly earlier puberty was not found to worsen students’ quality of life scores when comparing them with a group maturing at a gender- and age-typical rate. Gender proved to be a significant predictor in all models, with only the Peers and Social Support dimension yielding results in favor of girls. The inference of an association with age varies in subsequent models. Only the variation in the Autonomy and Parent Relations index shows no relationship with age. Pupils in the youngest age group, compared to the oldest group, rated their well-being significantly better in the Physical Well-Being and Psychological Well-Being dimensions but worse in the School Environment dimension. The comparison between the second and third groups provides an opportunity to assess changes in secondary schools. First-year students scored significantly better than third-year students from the same schools on the Physical Well-Being and Peers and Social Support dimensions and worse on the Psychological Well-Being dimension. The model estimated for the Psychological Well-Being index achieved the best goodness-of-fit coefficient (Table 4).

It is notable that, compared with the post hoc analysis based on the non-parametric K-W test, in many cases the inference of differences between groups changes if we adjust the analyses for other factors. The results of the detailed analyses according to the GLMs are included in the electronic Supplementary Materials (Table S3). There were no confirmed differences in Physical Well-Being scores or in Peers and Social Support scores. Regarding Psychological Well-Being, in many respects the inference is similar. However, there is no significant difference between those rating pubertal timing as typical and somewhat delayed. In the Autonomy and Parent Relations dimension, after adjusting for other factors, the difference between pubertal timing as normal and much earlier was reduced. The poorer perception of the school environment mainly applies to students maturing later than their same-sex peers. Previous simple comparisons indicated differences between eight pairs of groups.

**Figure 1:**
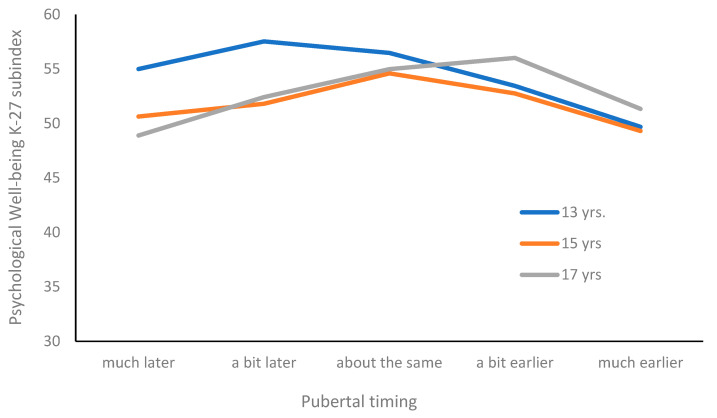
Psychological well-being and pubertal timing: age differences.

Additionally, several significant 2-way interactions were observed between pubertal timing and sex, grade, or contextual factors as predictors of various aspects of students’ quality of life (Figure 1).

**Figure 2:**
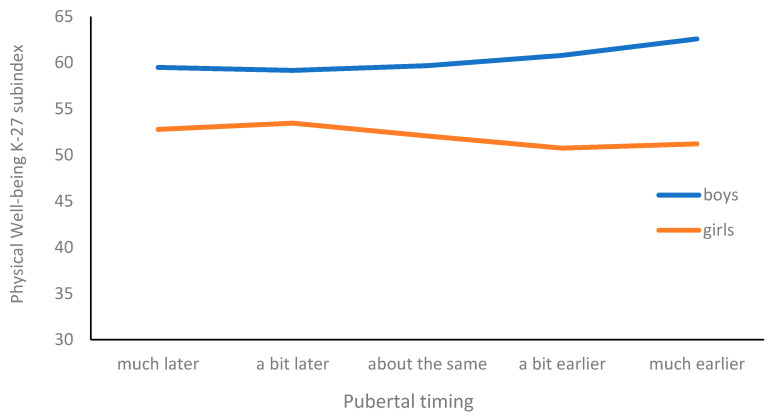
Physical well-being and pubertal timing: sex differences.

The alternative model estimated for the Psychological Well-Being dimension showed a strong interaction between pubertal timing and grade (*p* < 0.001). Younger adolescents reported higher scores compared to older age groups when pubertal timing was perceived as later or typical. However, in the case of accelerated maturation, these age differences tend to diminish (see Figure 2). Furthermore, this model also showed a significant interaction between pubertal timing and quality of life scores on the Psychological Well-being dimension (*p* = 0.021) and Autonomy and Parent Relations dimension (*p* = 0.043).

The interaction of pubertal timing with gender is best visible for the Physical Well-Being dimension (*p* = 0.001). After the introduction of this 2-way interaction, the inference on the significance of the pubertal timing main effect changed. The difference in favor of boys widens in the group assessing puberty as much earlier than their peers (Figure 2).

## 4. Discussion

This study presented a profile of health-related quality of life among 9411 Polish adolescents, based on their self-perceived pubertal timing. The widely used KIDSCREEN-27 questionnaire was employed, which allows for the assessment of five dimensions of physical and psychosocial well-being. Deviations from typical pubertal timing served as the starting point for exploring its impact on quality of life. The overall HRQL profile, based on standardized index scores, indicated the highest ratings in the Autonomy and Parent Relations dimension and the lowest in the School Environment dimension. Associations with pubertal timing were observed across all five dimensions, with the strongest relationship in Psychological Well-Being and the weakest in Physical Well-Being. However, the inference about the association of HRQL with perceived pubertal timing changes if we adjust the analyses for age, gender, and selected individual and contextual factors. The strongest association with Psychological Well-Being persists, and the association with the dimensions Physical Well-Being and Peers and Social Support is no longer significant. It is also worth noting that quality-of-life scores tended to be especially low among adolescents who perceived their pubertal timing as much earlier or much later than their peers, as opposed to those with only slight deviations from the perceived norm among their peers.

Early maturation has been identified in the literature as a significant risk factor for reduced HRQL when compared to typical or late pubertal timing [27]. Previous studies have found associations between earlier maturation and poorer sleep quality, mental health difficulties, poorer physical health, and lower overall quality of life [28]. Furthermore, adolescents who matured early and experienced negative influences in their microsystem (characterized by high levels of negative factors and low levels of positive factors) exhibited a higher incidence of difficulties, including both behavioral issues and depression—with the latter observed only among girls [29].

There is a significantly larger body of research on the consequences of early pubertal timing than there is for late timing [30]. Social dynamics within peer groups may exacerbate the challenges faced by late-maturing adolescents. The “off-time” hypothesis suggests that both early- and late-maturing adolescents are likely to experience higher levels of emotional difficulties compared to their peers who mature at a “normative” or typical pace. Changes that occur at socially expected ages are generally seen as predictable and culturally acceptable [31].

Particular attention should be given to the mental health dimension as a critical component of HRQL. Our study found that psychological functioning was poorer among both early- and late-maturing individuals. Other studies support this finding, indicating that adolescents who enter puberty earlier than same-age, same-sex peers are at an increased risk for mental health disorders [32,33]. However, some research points to an elevated risk of depression and anxiety in boys who experience delayed puberty. For example, one study found that depression was associated with early pubertal timing in girls and late timing in boys [34]. In contrast, however, a 2017 meta-analysis found no significant association between late pubertal timing and mental health disorders in either boys or girls [33].

We highlighted gender- and age-dependent differences in perceptions of pubertal timing. Younger adolescents may have less stable reference points for comparison, making their perceptions more polarized. Furthermore, our findings revealed age-related differences in psychological well-being. These differences were more pronounced among adolescents with later or typical pubertal timing, whereas they tended to diminish among those who matured early. It is possible that in the younger age group (13-year-olds), delayed maturation is less distressing, as developmental differences are less noticeable in peer interactions at that stage. As a result, late-maturing adolescents may not yet experience a strong sense of being different from their peers. In contrast, among older age groups (15 and 17 year olds), delayed maturation may present greater psychosocial challenges. This may help explain some of the observed discrepancies in the results, especially given that earlier research has shown that the absence of visible physical maturation can lead to feelings of inadequacy and increased anxiety when among peers who have already gone through puberty [35].

Regarding sex differences, we also found that girls with early pubertal timing rated their physical well-being lower, whereas for boys, negative effects were primarily associated with delayed maturation. Moreover, the association of psychological well-being with pubertal timing only became apparent in the interaction with gender. Late-maturing boys may face heightened peer pressure, stigma related to underdeveloped physiques, and lower social status in adolescent hierarchies, which may in turn affect self-esteem and mental health. These results are consistent with the hypothesis that during puberty, sex differences become more pronounced and may significantly influence participation in sports—an important factor contributing to physical well-being, as measured by the first KIDSCREEN-27 subindex. Research has shown that pubertal changes in boys are associated with increased muscle mass, anaerobic power, and strength [36], which are generally seen as advantageous for sports performance. In contrast, pubertal changes in girls often include increased fat mass, widening of the hips, and breast development, which may pose barriers to athletic achievement and participation in physical activities [37]. On the other hand, a systematic review of 78 studies found that early maturation was associated with lower levels of physical activity and greater sedentary behavior [38]. In our study, the physical dimension of HRQL was strongly associated with physical activity, fitness, and vitality. Regardless of the influence of physiological and biological factors, the effects of puberty on physical well-being understood in this way can be moderated and/or mediated by endogenous or exogenous psychosocial factors, which are strongly evident in girls [39,40].

Our study also found an association between the school environment and adolescents’ quality of life. Wang et al. [5] using data from the Fragile Families and Child Wellbeing Study—a study conducted in large U.S. cities—emphasized that adolescents require social connections in their immediate surroundings—including the school environment—which significantly influence their health behaviors and overall well-being. Young people spend a substantial portion of their lives at school, and the individuals in that setting often become central figures in their lives. A study by Carter et al. [41] showed that primary school teachers have varying social and academic expectations for girls at different stages of maturation, often perceiving early-maturing girls as more likely to face future difficulties. Similar findings were reported by Cavanagh et al., [42] who found that girls who experienced early menarche had more academic challenges during the transition to high school, which contributed to lower performance and increased dropout rates toward the end of schooling.

When considering the broader developmental environment, it is also important to address the role of the family context. Research indicates that early-maturing adolescents who perceived their relationship with their parents as insecure (i.e., insecure attachment) reported higher levels of depression and anxiety than their peers—whether early-, typical-, or late-maturing—who had secure attachments to their parents [43].

### Strengths and Limitations

One of the key strengths of this study is its broad population coverage within a defined administrative region—representing approximately 30% of the student population in that area. To the best of our knowledge, this is the first study of its kind in Poland to analyze the relationship between pubertal timing and quality of life. An additional strength is the use of the well-established KIDSCREEN questionnaire, which was adapted for implementation in Poland already at the development stage.

One limitation of this study is its cross-sectional design. This type of study does not allow for observations of changes over time nor for following the development trajectory. This highlights the need for future longitudinal research to track changes in quality of life over the years and gain a deeper understanding of the long-term effects of both early and late pubertal timing on adolescent development. This type of longitudinal study was carried out in Spain using the full version of KIDSCREEN-52, as a continuation of the research on which development of these tools are based [44].

Secondly, although we considered psychosocial factors in the models presented above, other factors that may influence HRQL change trajectories in the context of biological age and subjectively and objectively assessed maturation may also be considered in the future. An example of such analysis is a Norwegian 4-year prospective study also based on the KIDSCREEN-27 tool which included a group of adolescents similar in age but less numerous (N = 211). It demonstrated that stress, loneliness, and pain have a significant negative impact on HRQOL changes over time, whereas self-esteem and self-efficacy have a significant positive impact [45].

Finally, data on the onset of puberty were obtained through self-reported assessments. Our single-item assessment is an alternative to more complex tools that are worth including in subsequent studies, particularly in longitudinal studies. An example of this is the Puberty Development Scale (PDS), which contains three questions common to both sexes and two questions specific to boys and girls, and has a defined system for interpreting the results [46]. Scales of this type are very useful in determining the current state of development, but they do not refer to a self-assessment of the overall development process retrospectively, especially its onset evaluated from a longer perspective. We did not use Tanner staging to assess pubertal development. The objective method is, of course, more accurate in determining the timing of puberty but due to its sensitive nature is not suitable for large-scale epidemiological or sociological studies conducted in schools and can be easily rejected by adolescent respondents [47]. However, existing research suggests that adolescents’ self-assessment of pubertal stage correlates strongly with hormone levels and supports the validity of this method in scientific studies [48]. This limitation also meant that we could not compare adolescents’ subjective perceptions of pubertal timing with objective assessments, as such comparisons are not feasible in non-clinical settings or in large-scale samples. Importantly, perceived pubertal timing may shape psychosocial adjustment independently of objective biological markers. Adolescents form self-concepts and interpret peer reactions based on how they view their own development, which can influence behavior and mental health even when this perception is inaccurate. These findings suggest that preventive interventions may benefit from more comprehensively addressing adolescents’ perceptions of their own maturity, not only their actual biological development. In cases where there is a discrepancy between perceived and actual pubertal timing, adolescents may face a range of health risks, including an increased likelihood of engaging in harmful behaviors and difficulties with social adjustment [49]. This may represent an important direction for future research.

## 5. Conclusions

Our findings demonstrate that pubertal timing can be an important factor influencing adolescents’ psychological well-being. Both early and late maturation are associated with lower quality of life compared to typical pubertal timing. This issue deserves the attention of pediatricians and family physicians involved in preventive care, as well as school staff, particularly school psychologists and educational counselors. This is especially important, given that students whose pubertal timing differs from that of their peers tend to function less well in the school environment. The role of parents also remains crucial; by fostering secure relationships, they can help their children navigate this transitional period more smoothly. Moreover, considering the sex differences in how pubertal timing affects well-being, it is essential that future support programs be tailored to the specific needs of boys and girls at different stages of development. In practical terms, these findings suggest the need for school-based interventions, such as teacher training programs to enhance awareness of pubertal timing as an important factor influencing adolescents’ health-related quality of life. Additionally, screening initiatives that incorporate students’ subjective perceptions of their pubertal development could help identify those who may benefit from additional support. Such efforts may facilitate early response and promote well-being among adolescents experiencing either early or late maturation.

## Figures and Tables

**Table 1:** Mean KIDSCREEN-27 standardized (0–100) indices in a total sample and by sex and grade.

	KIDSCREEN-27 Standardized Score (0–100)				
	Physical Well-Being N = 9322	Psychological Well-Being N = 9147	Autonomy and Parent Relations N = 9180	Peers and Social Support N = 9277	School Environment N = 9276
Total	55.93 ± 21.95	54.09 ± 21.46	60.31 ± 21.96	58.80 ± 24.74	43.88 ± 21.32
Sex					
Boys	62.75 ± 21.72	59.67 ± 21.09	63.29 ± 22.41	58.24 ± 25.33	45.81 ± 22.45
Girls	50.26 ± 20.46	49.43 ± 20.65	57.84 ± 21.27	59.26 ± 24.23	42.28 ± 20.20
M-W test	Z = −27.64	Z = −23.27	Z = −12.72	Z = −1.65	Z = −8.14
*p*	<0.001	<0.001	<0.001	0.099	<0.001
Grade					
K-7	59.93 ± 21.78	56.44 ± 21.65	61.25 ± 22.34	58.34 ± 25.45	43.40 ± 21.85
K-9	53.98 ± 21.77	52.77 ± 21.41	60.19 ± 21.77	60.43 ± 24.19	44.96 ± 21.07
K-11	51.73 ± 21.30	51.87 ± 20.78	58.76 ± 21.48	56.92 ± 24.14	42.94 ± 20.64
K-W test	H = 238.89	H = 87.86	H = 22.74	H = 29.29	H = 15.96
*p*	<0.001	<0.001	<0.001	<0.001	<0.001

**Table 2:** Self-perceived pubertal timing.

	Total *N*/%	Pubertal Timing Comparing to Peers at Same Age (%)				
		“Much Later”	“A Bit Later”	“About the Same”	“A Bit Earlier”	“Much Earlier”
Total	9329/100.0%	5.8	16.7	49.0	20.0	8.5
Sex						
Male	4245/45.5%	7.2	18.5	46.9	19.6	7.9
Female	5084/54.5%	4.6	15.3	50.7	20.3	9.1
*χ*^2^ = 52.256; *df* = 4; *p* < 0.001						
Age category						
13 yrs	3818/40.9%	7.1	18.0	45.8	20.8	7.1
15 yrs	3465/37.1%	4.8	15.9	51.7	19.1	4.8
17 yrs	2046/21.9%	4.9	15.7	50.3	20.0	4.9
*χ*^2^ = 42.776; *df* = 8; *p* < 0.001						

**Table 3:** Mean KIDSCREEN-27 indices by pubertal timing (standardized scores on a 0–100 scale; higher scores indicate better HRQL).

Pubertal Timing	KIDSCREEN-27 Standardized Score (0–100)				
	Physical Well-Being N = 9245	Psychological Well-Being N = 9074	Autonomy and Parent Relations N = 9104	Peers and Social Support N = 9199	School Environment N = 9197
Total	55.94 ± 21.95	54.10 ± 21.46	60.32 ± 21.92	58.83 ± 24.75	43.90 ± 21.31
“Much later”	54.11 ± 24.89	49.26 ± 22.59	53.97 ± 25.74	53.79 ± 29.20	38.48 ± 23.76
“A bit later”	55.65 ± 21.90	53.38 ± 20.67	57.85 ± 21.51	56.93 ± 24.30	41.93 ± 21.13
“About the same”	57.25 ± 21.10	56.93 ± 20.58	63.19 ± 20.87	60.88 ± 23.59	46.27 ± 20.72
“A bit earlier”	54.91 ± 22.13	52.47 ± 21.66	58.92 ± 21.53	58.12 ± 24.83	43.18 ± 21.02
“Much earlier”	52.59 ± 23.71	46.33 ± 23.55	56.01 ± 24.21	55.91 ± 27.45	39.54 ± 22.09
K-W test	*H* = 38.32	*H* = 199.36	*H* = 158.70	*H* = 61.54	*H* = 143.72
*p*-value	*p* < 0.001	*p* < 0.001	*p* < 0.001	*p* < 0.001	*p* < 0.001

**Table 4:** Estimation of the general linear (GLM) model * for KIDSCREEN-27 indices (N = 8647).

Independent Variables	Dependent Variable									
	K-27: Physical Well-Being		K-27: Psychological Well-Being		K-27: Autonomy and Parent Relations		K-27: Peers and Social Support		K-27: School Environment	
	B	*p*	B	*p*	B	*p*	B	*p*	B	*p*
Model constant	14.51	0.000	7.44	0.000	22.22	0.000	20.60	0.000	7.36	0.000
Gender—boys	8.02	0.000	4.80	0.000	1.62	0.000	−7.00	0.000	−2.01	0.000
Grade—K-7	5.70	0.000	1.21	0.008	0.34	0.509	−0.91	0.143	−2.21	0.000
Grade—K-13	1.06	0.035	−1.04	0.022	−0.10	0.846	2.73	0.000	0.89	0.083
PT—“much later”	−0.30	0.726	−2.93	0.000	−3.93	0.000	−0.26	0.805	−2.46	0.004
PT—“bit later”	0.22	0.690	−0.99	0.041	−2.60	0.000	−0.36	0.585	−1.63	0.003
PT—“a bit earlier”	−0.11	0.824	−1.85	0.000	−1.75	0.001	0.23	0.712	−0.41	0.419
PT—“much earlier”	0.80	0.253	−5.63	0.000	−1.39	0.051	0.41	0.640	−1.53	0.033
K-27: Physical Well-Being	-	-	0.29	0.000	0.05	0.000	0.17	0.000	0.15	0.000
K-27: Psychological Well-Being	0.35	0.000	-	-	0.28	0.000	0.22	0.000	0.26	0.000
K-27: Autonomy and Parent Relations	0.05	0.000	0.22	0.000	-	-	0.27	0.000	0.22	0.000
K-27: Peers and Social Support	0.11	0.000	0.12	0.000	0.18	0.000	-	-	0.05	0.000
K-27: School Environment	0.15	0.000	0.21	0.000	0.22	0.000	0.07	0.000	-	-
Adjusted R-squared	0.365		0.460		0.341		0.237		0.299	

* PT: Pubertal timing; K-27: KIDSCREEN-27 indices as continuous variables; B—regression parameter; girls, K-11 and PT—“about the same” as reference categories; p - statistical significance

## Data Availability

The data presented in this study are not publicly available due to privacy and ethical restrictions.

## Supplementary Materials

The following supporting information can be downloaded at https://www.mdpi.com/article/10.3390/pediatric17030069/s1, Table S1: Correlation between KIDSCREEN-27 indices; Table S2: Pairwise comparison of KIDSCREEN-27 indices in post hoc analysis for Kruskal-Wallis test (significance level); Table S3. Pairwise comparison of KIDSCREEN-27 scores based on the GLM model adjusted for sex, grade and other KIDSCREEN-27 domains (significant results bolded).
