# Supplementary material for: Pubertal Timing and Health-Related Quality of Life—A Cross-Sectional Study of Polish Adolescents
Source: Pediatr Rep. 2025 Jun 18;17(3):69. doi: 10.3390/pediatric17030069 (PMC12195768; doi:10.3390/pediatric17030069)
Supplement: Supplementary file 1 [file pediatrrep-17-00069-s001.zip › pediatrrep-3653810-supplementary.pdf]

Supplementary Material

Table S1. Correlations between KIDSCREEN-27 indices.

KIDSCREEN-27 domains*	**N**	**Pearson's r**	**P**	**95% CI**
D1-D2	9074	0.536	0.000	0.521-0.550
D1-D3	9097	0.357	0.000	0.339-0.375
D1-D4	9193	0.314	0.000	0.295-0.332
D1-D5	9194	0.377	0.000	0.360-0.395
D2-D3	8939	0.498	0.000	0.482-0.513
D2-D4	9025	0.380	0.000	0.362-0.397
D2-D5	9022	0.472	0.000	0.455-0.488
D3-D4	9087	0.399	0.000	0.382-0.416
D3-D5	9073	0.429	0.000	0.412-0.445
D4-D5	9276	0.299	0.000	0.280-0.318

*D1—Physical Well-Being; D2—Psychological Well-Being; D3—Autonomy and Parent Relations; D4—Peers and Social Support; D5—School Environment.

Table S2. Pairwise comparisons of KIDSCREEN-27 indices using a post hoc analysis based on the Kruskal–Wallis test (significance levels).

**Sample 1—Sample 2**	**Physical Well-being**	**Psychological Well-being**	**Autonomy and Parent Relations**	**Peers and Social Support**	**School Environment**
Much earlier—A bit earlier	0.032	<0.001	0.011	0.062	<0.001
Much earlier—Much later	0.069	0.037	0.267	0.290	0.415
Much earlier—A bit later	0.002	<0.001	0.148	0.460	0.005
Much earlier—About the same	<0.001	<0.001	<0.001	<0.001	<0.001
A bit earlier—Much later	0.827	<0.001	0.001	0.005	<0.001
A bit earlier—A bit later	0.222	0.166	0.195	0.174	0.160
A bit earlier—About the same	<0.001	<0.001	<0.001	<0.001	<0.001
Much later—A bit later	0.532	<0.001	0.013	0.069	0.001
Much later—About the same	0.027	<0.001	<0.001	<0.001	<0.001
A bit later—About the same	0.018	<0.001	<0.001	<0.001	<0.001

Table S3. Pairwise comparisons of KIDSCREEN-27 scores based on the GLM model adjusted for sex, grade, and other KIDSCREEN-27 domains (significant results shown in bold).

a) Physical Well-Being Domain

Perceived Puberty Timing		Difference Between Means	SE	p	95% CI	
					Lower	Upper
Much later	A bit later	-0.488	0.996	1.000	-3.284	2.308
	A bit earlier	-0.139	0.974	1.000	-2.873	2.596
	Much earlier	-1.314	1.094	1.000	-4.386	1.758
	About the same	-0.110	0.916	1.000	-2.683	2.463
A bit later	A bit earlier	0.350	0.659	1.000	-1.500	2.199
	Much earlier	-0.826	0.829	1.000	-3.154	1.502
	About the same	0.378	0.566	1.000	-1.210	1.966
A bit earlier	Much earlier	-1.176	0.802	1.000	-3.429	1.077
	About the same	0.028	0.524	1.000	-1.442	1.499
Much earlier	About the same	1.204	0.730	0.992	-0.846	3.254

F test = 0.779; df = 4; p = 0.538.

b) Psychological Well-Being Domain

Perceived Puberty Timing		Difference Between Means	SE	p	95% CI	
					Lower	Upper
Much later	A bit later	-1.937	0.832	0.199	-4.273	0.399
	A bit earlier	-1.077	0.814	1.000	-3.363	1.209
	Much earlier	2.696	0.924	**0.035**	0.101	5.290
	About the same	-2.931	0.764	**0.001**	-5.077	-0.785
A bit later	A bit earlier	0.860	0.565	1.000	-0.727	2.447
	Much earlier	4.633	0.716	**0.000**	2.623	6.642
	About the same	-0.994	0.487	0.413	-2.361	0.374
A bit earlier	Much earlier	3.772	0.693	**0.000**	1.827	5.718
	About the same	-1.854	0.451	**0.000**	-3.121	-0.587
Much earlier	About the same	-5.626	0.631	**0.000**	-7.399	-3.853

F test = 22.564; df = 4; p < 0.001.

c) Autonomy and Parent Relations Domain

Perceived Puberty Timing		**Difference Between Means**	**SE**	**p**	**95% CI**	
					**Lower**	**Upper**
Much later	A bit later	-1.329	0.936	1.000	-3.956	1.299
	A bit earlier	-2.178	0.915	0.173	-4.748	0.391
	Much earlier	-2.541	1.039	0.145	-5.458	0.376
	About the same	-3.931	0.859	**0.000**	-6.343	-1.519
A bit later	A bit earlier	-0.850	0.635	1.000	-2.634	0.934
	Much earlier	-1.212	0.806	1.000	-3.477	1.052
	About the same	-2.602	0.547	**0.000**	-4.138	-1.067
A bit earlier	Much earlier	-0.363	0.780	1.000	-2.554	1.829
	About the same	-1.752	0.508	**0.006**	-3.178	-0.327
Much earlier	About the same	-1.390	0.713	0.513	-3.392	0.612

F test = 10.056; df = 4; $p < 0.001$.

d) Peers and Social Support Domain

Perceived Puberty Timing		**Difference Between Means**	**SE**	**p**	**95% CI**	
					**Lower**	**Upper**
Much later	A bit later	0.105	1.137	1.000	-3.088	3.298
	A bit earlier	-0.486	1.112	1.000	-3.609	2.637
	Much earlier	-0.663	1.263	1.000	-4.209	2.882
	About the same	-0.258	1.045	1.000	-3.192	2.676
A bit later	A bit earlier	-0.591	0.772	1.000	-2.759	1.577
	Much earlier	-0.768	0.980	1.000	-3.520	1.983
	About the same	-0.363	0.665	1.000	-2.231	1.505
A bit earlier	Much earlier	-0.177	0.948	1.000	-2.840	2.485
	About the same	0.228	0.617	1.000	-1.505	1.961
Much earlier	About the same	0.405	0.867	1.000	-2.028	2.839

F test = 0.231; df = 4; $p = 0.921$.

e) School Environment Domain

Perceived Puberty Timing		**Difference Between Means**	**SE**	**p**	**95% CI**	
					**Lower**	**Upper**
Much later	A bit later	-0.836	0.937	1.000	-3.468	1.796
	A bit earlier	-2.051	0.917	0.253	-4.625	0.523
	Much earlier	-0.936	1.041	1.000	-3.859	1.987
	About the same	-2.462	0.861	**0.043**	-4.880	-0.045
A bit later	A bit earlier	-1.215	0.636	0.564	-3.002	0.572
	Much earlier	-0.100	0.808	1.000	-2.369	2.168
	About the same	-1.626	0.548	**0.030**	-3.166	-0.087
A bit earlier	Much earlier	1.114	0.782	1.000	-1.080	3.309
	About the same	-0.411	0.509	1.000	-1.840	1.017
Much earlier	About the same	-1.526	0.714	0.327	-3.531	0.480

F test = 4.140; df = 4; p = 0.00.
